# Supplementary material for: Modeling glioblastoma heterogeneity as a dynamic network of cell states
Source: Mol Syst Biol. 2021 Sep 16;17(9):e10105. doi: 10.15252/msb.202010105 (PMC8444284; doi:10.15252/msb.202010105)
Supplement: Supplementary file 6 — Source Data for Figure 5 [file MSB-17-e10105-s004.zip › Figure5A_sourcedata/GSEA_3071/hallmarks_stateII.GseaPreranked.1623742536692/HALLMARK_PROTEIN_SECRETION.html]

Details for gene set HALLMARK\_PROTEIN\_SECRETION[GSEA]

|  || Dataset | state23071 |
| Phenotype | NoPhenotypeAvailable |
| Upregulated in class | na\_neg |
| GeneSet | HALLMARK\_PROTEIN\_SECRETION |
| Enrichment Score (ES) | -0.20570709 |
| Normalized Enrichment Score (NES) | -0.8680491 |
| Nominal p-value | 0.65309733 |
| FDR q-value | 0.81347483 |
| FWER p-Value | 1.0 |
Table: GSEA Results Summary

  

Fig 1: Enrichment plot: HALLMARK\_PROTEIN\_SECRETION      
 Profile of the Running ES Score & Positions of GeneSet Members on the Rank Ordered List

  

| PROBE | GENE SYMBOL | GENE\_TITLE | RANK IN GENE LIST | RANK METRIC SCORE | RUNNING ES | CORE ENRICHMENT || 1 | EGFR |  |  | 198 | 0.874 | 0.0139 | No |
| 2 | CAV2 |  |  | 344 | 0.714 | 0.0293 | No |
| 3 | CTSC |  |  | 833 | 0.489 | -0.0557 | No |
| 4 | AP2S1 |  |  | 871 | 0.479 | -0.0305 | No |
| 5 | ZW10 |  |  | 958 | 0.457 | -0.0189 | No |
| 6 | SSPN |  |  | 1081 | 0.421 | -0.0187 | No |
| 7 | ANP32E |  |  | 1334 | 0.371 | -0.0541 | No |
| 8 | RER1 |  |  | 1370 | 0.362 | -0.0368 | No |
| 9 | LMAN1 |  |  | 1605 | 0.323 | -0.0713 | No |
| 10 | TMX1 |  |  | 1703 | 0.310 | -0.0729 | No |
| 11 | AP3S1 |  |  | 2014 | 0.271 | -0.1297 | No |
| 12 | RAB22A |  |  | 2324 | -0.262 | -0.1870 | Yes |
| 13 | AP1G1 |  |  | 2395 | -0.270 | -0.1849 | Yes |
| 14 | ARFGEF1 |  |  | 2425 | -0.273 | -0.1724 | Yes |
| 15 | SNAP23 |  |  | 2452 | -0.277 | -0.1590 | Yes |
| 16 | STX16 |  |  | 2468 | -0.279 | -0.1427 | Yes |
| 17 | STX7 |  |  | 2566 | -0.296 | -0.1453 | Yes |
| 18 | GOLGA4 |  |  | 2582 | -0.298 | -0.1277 | Yes |
| 19 | SNX2 |  |  | 2650 | -0.308 | -0.1221 | Yes |
| 20 | COPB2 |  |  | 2661 | -0.310 | -0.1024 | Yes |
| 21 | RAB9A |  |  | 2671 | -0.312 | -0.0823 | Yes |
| 22 | LAMP2 |  |  | 2724 | -0.321 | -0.0721 | Yes |
| 23 | IGF2R |  |  | 2736 | -0.324 | -0.0516 | Yes |
| 24 | CLN5 |  |  | 2782 | -0.332 | -0.0389 | Yes |
| 25 | SCRN1 |  |  | 3051 | -0.386 | -0.0772 | Yes |
| 26 | COPE |  |  | 3153 | -0.414 | -0.0724 | Yes |
| 27 | TMED10 |  |  | 3179 | -0.423 | -0.0482 | Yes |
| 28 | AP2M1 |  |  | 3272 | -0.448 | -0.0388 | Yes |
| 29 | RAB5A |  |  | 3316 | -0.462 | -0.0163 | Yes |
| 30 | PAM |  |  | 3685 | -0.671 | -0.0588 | Yes |
| 31 | CLCN3 |  |  | 3734 | -0.711 | -0.0197 | Yes |
| 32 | GNAS |  |  | 4036 | -1.522 | 0.0152 | Yes |
Table: GSEA details [plain text format]

  

Fig 2: HALLMARK\_PROTEIN\_SECRETION: Random ES distribution      
 Gene set null distribution of ES for **HALLMARK\_PROTEIN\_SECRETION**
